# Examining the effect of *Withania somnifera* supplementation on muscle strength and recovery: a randomized controlled trial

**DOI:** 10.1186/s12970-015-0104-9

**Published:** 2015-11-25

**Authors:** Sachin Wankhede, Deepak Langade, Kedar Joshi, Shymal R. Sinha, Sauvik Bhattacharyya

**Affiliations:** Sports Medicine, SrimatiKashibaiNavale Medical College, Pune, India; Department of Pharmacology, BVDU Dental College & Hospital, Navi Mumbai, India; Department of Pharmacology, BharatiVidyapeeth Medical College & Hospital, Sangli, India; Department of Clinical Pharmacology, Grant Government Medical College, SirJamshedjeeJeejeebhoy Group of Hospitals, Mumbai, India; Department of Pharmaceutical Technology, NSHM Knowledge Campus, 124 B.L. Saha Road, Kolkata, 700053 India

**Keywords:** Ashwagandha, Adaptogen herbs, Muscle, Muscle strength, Muscle mass, Testosterone, Body fat, Creatine kinase

## Abstract

**Background:**

*Withania somnifera* (a*shwagandha*) is a prominent herb in Ayurveda. This study was conducted to examine the possible effects of ashwagandha root extract consumption on muscle mass and strength in healthy young men engaged in resistance training.

**Methods:**

In this 8-week, randomized, prospective, double-blind, placebo-controlled clinical study, 57 young male subjects (18–50 years old) with little experience in resistance training were randomized into treatment (29 subjects) and placebo (28 subjects) groups. Subjects in the treatment group consumed 300 mg of ashwagandha root extract twice daily, while the control group consumed starch placebos. Following baseline measurements, both groups of subjects underwent resistance training for 8 weeks and measurements were repeated at the end of week 8. The primary efficacy measure was muscle strength. The secondary efficacy measures were muscle size, body composition, serum testosterone levels and muscle recovery. Muscle strength was evaluated using the 1-RM load for the bench press and leg extension exercises. Muscle recovery was evaluated by using serum creatine kinase level as a marker of muscle injury from the effects of exercise.

**Results:**

Compared to the placebo subjects, the group treated with ashwagandha had significantly greater increases in muscle strength on the bench-press exercise (Placebo: 26.4 kg, 95 % CI, 19.5, 33.3 vs. Ashwagandha: 46.0 kg, 95 % CI 36.6, 55.5; *p* = 0.001) and the leg-extension exercise (Placebo: 9.8 kg, 95 % CI, 7.2,12.3 vs. Ashwagandha: 14.5 kg, 95 % CI, 10.8,18.2; *p* = 0.04), and significantly greater muscle size increase at the arms (Placebo: 5.3 cm^2^, 95 % CI, 3.3,7.2 vs. Ashwagandha: 8.6 cm^2^, 95 % CI, 6.9,10.8; *p* = 0.01) and chest (Placebo: 1.4 cm, 95 % CI, 0.8, 2.0 vs. Ashwagandha: 3.3 cm, 95 % CI, 2.6, 4.1; *p* < 0.001). Compared to the placebo subjects, the subjects receiving ashwagandha also had significantly greater reduction of exercise-induced muscle damage as indicated by the stabilization of serum creatine kinase (Placebo: 1307.5 U/L, 95 % CI, 1202.8, 1412.1, vs. Ashwagandha: 1462.6 U/L, 95 % CI, 1366.2, 1559.1; *p* = 0.03), significantly greater increase in testosterone level (Placebo: 18.0 ng/dL, 95 % CI, -15.8, 51.8 vs. Ashwagandha: 96.2 ng/dL, 95 % CI, 54.7, 137.5; *p* = 0.004), and a significantly greater decrease in body fat percentage (Placebo: 1.5 %, 95 % CI, 0.4 %, 2.6 % vs. Ashwagandha: 3.5 %, 95 % CI, 2.0 %, 4.9 %; *p* = 0.03).

**Conclusion:**

This study reports that ashwagandha supplementation is associated with significant increases in muscle mass and strength and suggests that ashwagandha supplementation may be useful in conjunction with a resistance training program.

## Background

Both the modern medical literature and traditional Ayurveda writings report many potential health benefits of the Ashwagandha herb (*Withania somnifera*, also known as Indian Ginseng or Winter Cherry) under the rubrics of anti-stress effects, neuroprotective effects, immunomodulatory effects, and rejuvenating effects, via the herb's interplay with the nervous system, the endocrine system, the cardiopulmonary system, the energy production system and the immune system including analgesic, antimicrobial, anti-inflammatory, anti-tumor, anti-stress, anti-diabetic, neuroprotective, immunoprotective and cardioprotective effects [1–7]. This paper focuses on ashwagandha as an ergogenic aid and is the first to present the results of a randomized, double-blind, placebo-controlled clinical study on ashwagandha’s effects, as an adjuvant to a resistance training program, multifariously on muscle strength, muscle hypertrophy, muscle recovery and body composition. We add to the broader literature on ashwagandha’s effect on physical performance. This literature has only a small set of published papers [8–10], which is surprising because traditional Ayurveda explicitly advocates the use of ashwagandha toward “bala”, which means “strength” in the Sanskrit language [11] .

Ashwagandha is a member of the family of herbs referred to as “adaptogens”. The term “adaptogen” is applied to a herb with phytonutrients that regulate metabolism when a body is perturbed by physical or mental stress, and help the body adapt by (a) normalizing system functions, (b) developing resistance to future such stress, and (c) elevating the body’s functioning to a higher level of performance [12]. The adaptogen family of herbs has many members, noteworthy among them being ashwagandha, rhodiola, ginseng, schisandra and maca [12]. Adaptogens are used commonly for stress relief, brain health, adrenal health and for ameliorating HPA-axis dysfunction. More recently, adaptogens have started to be used in sports supplements that aim to enhance physical fitness. Recent research has found adaptogens to be promising in this application domain [13–15]. However, the results in this literature are mixed and therefore more research is needed so that we have a better understanding of adaptogens as ergogenic aids [14]. This present study attempts to make a small step towards such an understanding.

A resistance training program consists of exercises that cause skeletal muscles to contract against external resistance. The body often responds to such programs with increased strength and correlated adaptations [16–19]. The present research work was motivated by the hypothesis that ashwagandha supplementation can increase some of these adaptations and gains, thereby serving as a useful adjuvant to a resistance training program. There are several rationales underlying this hypothesis: Studies in healthy normal adults demonstrated that ashwagandha improves muscular strength/coordination, and cardiorespiratory endurance [8–10]. Ashwagandha’s roots are classified as a “rasayana” (rejuvenator), and have been used toward promoting health and longevity, slowing the aging process, revitalizing the body and generally creating a sense of well-being [4, 20]. Ashwagandha has a wide range of pharmacological effects: it has anxiolytic, hypotensive, sedative, central nervous system, immunomodulatory, analgesic, anti-inflammatory, anti-tumor, anabolic, cardiopulmonary and antioxidant effects [4, 9, 21–27]. It also stimulates respiratory function, causing smooth muscle relaxation, and stimulates thyroid activity [3]. Studies in humans show that ashwagandha is well tolerated and is associated with decreases in cortisol [28], and increases in testosterone [29]. Research suggests that ashwagandha may reduce increases of blood urea nitrogen, lactic acid, corticosterone in response to stress [4], and also reduce the tendency of dopamine receptors in the brain to activate under stress [3, 5, 21]. Ashwagandha contains several active components, which may account for the various mechanisms of action by which it exerts its effects. These include steroidal lactones (withanolides, withaferins), saponins and alkaloids like isopelletierine and anaferine [5, 30].

The adaptogenic properties of ashwagandha raise the possibility of it being an effective ergogenic aid because the strain from exercise can be viewed as a form of stress, with enhanced human physical performance as the corresponding stress response upon ashwagandha supplementation. This study seeks to examine the hypothesis that ashwagandha supplementation may moderate the body’s adaptation in response to resistance training. While this hypothesis is rooted in traditional Ayurvedic medicine and previous studies, well designed clinical trials are clearly needed to test and characterize the effects. The present double-blind, randomized, placebo-controlled study in healthy adults will, it is hoped, help toward expanding scientific understanding in this domain.

## Methods

The study design, recruitment and methods were approved by all the authors’ institutes’ review boards on human subjects’ studies and followed the guidelines of the Declaration of Helsinki and Tokyo for humans.

### Subjects selection, incentives and participation

**Fig. 1:**
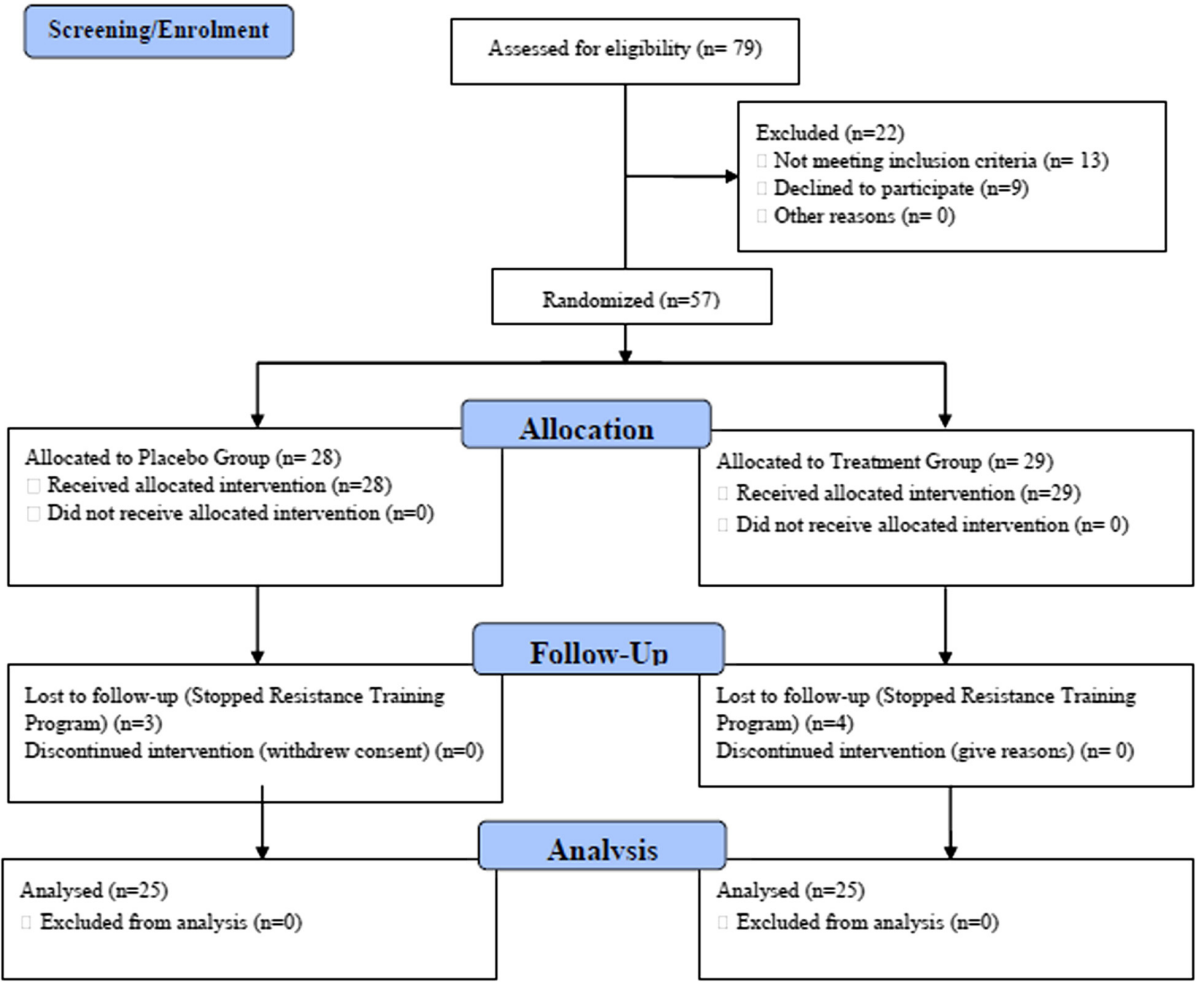
CONSORT schematic diagram

Healthy male subjects (18–50 years old) were recruited by use of fliers circulated in the vicinity of the gymnasium which served as the site of the training program. Subject enrollment, allocation, attrition, and analysis is summarized in Fig. 1. The purpose and protocol for the study was described to the subjects. The subjects had to sign a written consent for the study, agree to refrain from alcohol and tobacco during the study, and had to receive permission of their physician to participate. Subjects were excluded from the study if they met any of the following criteria: 1) taking any medication or steroids to enhance physical performance, 2) weight loss of >5 kg in the previous 3 months, 3) any history of drug abuse, smoking 10+ cigarettes day or consuming more than 14 grams of alcohol daily, 4) hypersensitivity to ashwagandha, 5) history of any orthopedic injury or surgery within the previous 6 months, 6) participation in other clinical studies during the previous 3 months, 7) any other conditions which the investigators judged problematic for participation in the study. Subjects were requested to refrain from using anti-inflammatory agents during the study and to report any ill effects from consuming the ashwagandha/ placebo.

Because participation in the study would require a significant amount of time allocation from the subjects, the recruiters offered as compensation one year of paid membership to the gym and three months of professional trainer support. As Fig. 1 indicates, 57 subjects were initially recruited for the study and 50 completed the study, including 25 in the ashwagandha group and 25 in the placebo group. For the ashwagandha treatment group and for the placebo group, the mean age ± standard deviation were 28 ± 8 years and 29 ± 9 years, respectively.

### Study design

This study was a prospective, double-blind, placebo-controlled parallel group study to measure the possible effects of ashwagandha extract on muscle strength/size, muscle recovery, testosterone level and body fat percentage in young males undergoing weight training. Adverse events were assessed by patient/ researcher reporting and the PGATT (Physicians Global Assessment of Tolerability to Therapy) form.

### The resistance training program

The resistance training program consisted of sets of exercises over major muscle groups in both the upper body and the lower body. Directions for the resistance training were obtained from publications of the National Strength and Conditioning Association (NSCA) [31–33]. Each subject in both groups was asked to come to a training session every other day, with one rest day per week, for three days per week. Every session began with a warm up consisting of five minutes of low-intensity aerobic exercise.

The subjects were instructed to perform, for each set, as many repetitions as they could until failure. The subjects were asked to go through the full range of motion and were demonstrated the proper technique for safe and effective weight lifting.

#### Exercise selection

The specific exercises and the number of sets in each session were as follows. For the first week, the subjects were asked to perform the barbell squat (2 sets), the leg extension (1 set), the seated leg curl (2 sets), the machine chest press (1 set), the barbell chest press (2 sets), the seated machine row (1 set), the one-arm dumbbell row (2 sets), the machine biceps curl (1 set), the dumbbell biceps curl (2 sets), the cable triceps press-down (2 sets), the dumbbell shoulder press (2 sets), and the straight-arm pull-down (2 sets). For the second week, the subjects were asked to perform the leg extension (1 set), the barbell squat (2 sets), the barbell chest press (3 sets), the seated leg curl (2 sets), the seated cable row (3 sets), dumbbell biceps curl (3 sets), the cable triceps press-down (3 sets), the dumbbell shoulder press (3 sets), and the straight-arm pull-down or lat pull-down (3 sets). After this two-week acclimatization phase, for the rest of the study, the subjects were asked to perform the barbell squat (3 sets), the leg extension (3 sets), the leg curl (2 sets), one chest exercise (flat, incline or decline press or fly, cable cross-over, 3 sets), one back exercise (rows, pull up, pull down or seated cable row, 3 sets), another chest exercise (3 sets), another back exercise (3 sets), one biceps exercise or one triceps exercise (curls or extensions, 3 sets), and one shoulder exercise (raises or presses, 3 sets).

#### How the target number of repetitions was chosen

The number of repetitions for the initial two weeks was set to be 15, which is a moderately high number (implying correspondingly lower weights), chosen to allow a subject’s body and neural system to get accustomed to strength training [31, 33]. The subsequent 6 weeks had a varying number of repetitions, akin to a rudimentary non-linear periodization programs, because these have been shown to induce greater adaptation and more gains in muscle strength and size [33]. The number of repetitions specified for each of the days in the training program is given in Table 1.

**Table 1:** The targeted number of repetitions over the course of the resistance training program

	Weeks 1–2	Weeks 3–4	Weeks 5–6	Weeks 7–8
Day 1	15	13	5	9
Day 2	15	9	13	5
Day 3	15	13	5	9
Day 4	15	9	13	5

#### How the load was chosen

The load was chosen to be such that the subject would reach the failure-point upon performing approximately the target number of repetitions (chosen as described in the preceding paragraph) when lifting that load. The corresponding load for an exercise was estimated on the basis of the 1RM prediction equations of Epley, Wathan and others [34].

### Treatments and dosing

The researchers engaged a local laboratory to fill cellulose-based vegetarian capsules with either 300 mg of starch or 300 mg of a high-concentration ashwagandha root extract, KSM-66, manufactured by Ixoreal BioMed, Los Angeles, California, USA. This extract was produced using a water-based process using no alcohol or solvents and is standardized to a 5 % concentration of withanolides as measured by HPLC. The two capsules were identical in appearance, weight, and texture. Both the control and ashwagandha groups received a bottle of 60 pills at start of study and at 4 weeks. The pill count at 4 weeks allowed for a compliance check. The subjects were instructed to store the capsules between 18 and 32 °C and to take the capsules twice a day, once shortly after awakening and again shortly before bed, for the 8 weeks of the study.

### Primary efficacy endpoint

The primary efficacy endpoint was muscle strength. Muscle strength is often measured by 1RM, the “one-repetition maximum”, which is specific to a certain person and a certain exercise movement and refers to the maximal load that a subject can lift for one movement cycle of the exercise [31, 35]. Measurements were made at the first day of training and again 2 days after the 8 week training ended. The equipment used machine models DPL0802 (bench press) and DSL0605 (leg extension), manufactured by Precor (Woodinville, Washington, USA The 1-RM measurement was done using a variant of the widely used Baechle-Earle-Wathen protocol, employing the multiple RM method [36–40]. To reduce measurement error in strength assessment, we were careful to ensure consistency in the range of motion and that each movement on the chest press and the leg extension was complete and in accordance with the guidelines of the NSCA.

### Secondary efficacy endpoints

The secondary efficacy endpoints related to serum testosterone level, muscle recovery and anthropometric factors capturing muscle size and body fat percentage.

#### Anthropometry

*Muscle size:* Muscle size was measured at 3 sites: the arm (flexed mid upper arm), chest (sternum at mid-tidal volume) and upper thigh (just inferior to gluteal fold). Measurements were done on the first day of the training period and 2 days after the last day of training. For the chest, we measured the girth, taken at the level of the middle of the sternum, with the tape passing under the arms and at the end of a normal expiration. For the thigh and arm, we assessed the maximal cross-sectional area (CSA) using the method of Moritani-DeVries, which is based on girth and skin-fold measurements [41–43]. The literature shows that the muscle CSA measures obtained by the Moritani-DeVries method are highly correlated with measures obtained by computer tomography or muscle biopsy, the gold standards for muscle CSA measurement [41–43]. Because of this high correlation, the across-time (Day 0 versus Day 56) or across-group (treatment versus placebo) comparisons on the basis of the Moritani-DeVries method are strongly indicative of the directionality and strength of the corresponding comparisons on the basis of the computer tomography. We chose to use the Moritani-DeVries method because it is less time-consuming and invasive than computer tomography or biopsy, and therefore less likely to discourage study participation.

#### Body fat percentage

Body fat percentage was calculated with a bioelectrical impedance method using machine with electrodes placed at the hand, wrist, foot, and ankle [44, 45]. Because bioelectrical impedance analysis based measurement of fat composition is known to be affected by extraneous factors like hydration level and temperature, we tried to maintain consistency in these factors by instructing the subjects to: 1) abstain from eating or drinking for 4 h before measurements, 2) urinate 30 min prior, 3) not engaged in exercise for 12 h prior and 4) not consume alcohol of caffeinated products [35, 45]. Body composition was measured two days after the first day of resistance training and again two days after the last day of resistance training.

#### Testosterone

Total blood testosterone serum levels were measured twice: once 2 days after the study commenced and again 2 days after the study ended. The blood draw was timed to be between two hours and three hours of each subject’s regular waking time, and prior to any substantial physical activity, in order to minimize the effects of the natural diurnal variation in testosterone level. The 20 ml blood draws were from an antecubital vein, punctured with a 20-gauge disposable needle connected to a Vacutainer tube. The blood serum samples were analyzed by an ELISA (enzyme-linked immunosorbent assay).

#### Muscle recovery

Resistance training frequently damages skeletal muscle tissue. Such damage can result in decreased muscle force production and performance in subsequent training sessions, thereby possibly reducing the extent of adaptation and gains from resistance training [46]. Muscle recovery refers to the reduction in exercise-induced muscle damage over time. The level of creatine kinase, a protein, in the blood is a commonly used measure of muscle damage because this protein is specific to muscle tissue [47]. When muscles are overexerted, the muscle filaments are damaged and become necrotic, thereby causing soluble proteins like creatine kinase to migrate from muscle tissue into the blood stream [48]. The body on its own repairs such damage over 1 to 10 days and serum creatine kinase returns to baseline levels [48]. A bout of exercise tends to produce less damage in muscle tissue when repeated in subsequent training sessions after the body gets accustomed to the exercise. This is because of adaptation and strengthening of the muscle tissue. Serum creatine kinase was measured at 24 h and at 48 h after the end of the first exercise session, and also at 24 h and at 48 h after the end of the last exercise session approximately 8 weeks later, from 20 ml blood draws using a 20-gauge disposable needle and a Vacutainer setup. The creatine kinase level was determined in a commercial laboratory using enzymatic analysis tracking nicotinamide adenine diphesphopyridine (NADPH). The increase in creatine kinase from the 24-h point to the 48-h point can be taken as a biomarker of recovery in that a smaller increase corresponds to faster stabilization of creatine kinase level and hence faster recovery of muscle tissue from exercise-induced damage.

#### Tolerability

The subjects were asked to report any adverse events experienced at any point in the study. We used the Physicians Global Assessment of Tolerability to Therapy (PGATT) form [49–51]. Subjects used a five-point scale to assess tolerability from “worst tolerability” (which corresponds to patients’ not being able to tolerate the drug at all) to “excellent tolerability” (which corresponds to no adverse effects and the patient being able to tolerate the drug excellently).

#### Statistical analysis

Assessment of statistical significance of continuous treatment effects was done using ANOVA with group identity (treatment versus placebo) as a factor. We used the Mann-Whitney test if the data were found to be not normally distributed. Frequencies of the tolerability scale values were compared using the chi-square test for contingency tables. The accepted level of significance was α = 0.05.

## Results

Tables 2, 3, 4, 5 and 6 compare the treatment group and the placebo group at baseline, at the start of the study and at the end of the 8 week study for the following 5 factors: testosterone level (ng/dL), muscle strength on the bench press 1-RM (Kg), muscle strength on the leg extension 1-RM (Kg), muscle size at thighs (cm^2^), muscle size at arms (cm^2^), muscle size at chest (cm), body fat percentage, muscle recovery in terms of creatine kinase levels change (U/L),

**Table 2:** Muscle strength

		Treatment group	Placebo group	Between group comparison
		Mean (SD)	Mean (SD)	(*p*-values)
	Sample size (n)	*n* = 25	*n* = 25	
Bench Press 1RM (Kg)	Pre intervention	33.21 (8.50)	31.35 (7.97)	0.44
	Post intervention	79.26 (25.90)	57.77 (16.48)	0.001
	Change	46.05 (23.00); 95 % CI: 36.56, 55.54**	26.42 (16.72); 95 % CI: 19.52, 33.32**	0.001
Leg Extension 1RM (Kg)	Pre intervention	27.89 (4.24)	25.22 (7.03)	0.11
	Post intervention	42.38 (10.80)**	34.98 (10.54)**	0.02
	Change	14.50 (9.04); 95 % CI: 10.76, 18.23**	9.77 (6.27); 95 % CI: 7.18, 12.35**	0.04

** = *p* < 0.001 within group comparison

**Table 3:** Muscle size

		Treatment group mean (SD)	Placebo group mean (SD)	Between group comparison
				(*p*-values)
	Sample size (n)	*n* = 25	*n* = 25	
Thigh (cm^2^)	Pre intervention	107.84 (24.61)	111.18 (17.15)	0.58
	Post intervention	116.56 (26.04)	117.40 (19.96)	0.9
	Change	8.71 (10.06); 95 % CI: 4.56, 12.87**	6.22 ( 8.76); 95 % CI: 2.61, 9.84*	0.36
Arm (cm^2^)	Pre intervention	51.96 (10.88)	53.13 (14.84)	0.75
	Post intervention	60.85 (13.23)	58.43 (17.66)	0.59
	Change	8.89 (4.71); 95 % CI: 6.95, 10.84**	5.30 ( 4.74); 95 % CI: 3.34, 7.25**	0.01
Chest (cm)	Pre intervention	101.40 (11.22)	101.16 (8.93)	0.93
	Post intervention	104.77 (11.09)	102.58 (8.76)	0.44
	Change	3.37 ( 1.89); 95 % CI: 2.59, 4.15**	1.43 (1.45); 95 % CI: 0.83, 2.02**	0.0002

* = *p* < 0.01; ** = *p* < 0.001 within group comparison

**Table 4:** Body fat percentage

	Treatment group	Placebo	Between group comparison
	Mean (SD)	Group mean (SD)	(*p*-values)
Sample size (n)	*n* = 25	*n* = 25	
Pre intervention	21.60 (3.91)	22.01 (3.37)	0.7
Post intervention	18.13 (3.13)**	20.48 (1.85)*	0.003
Change	−3.47 (3.58); 95 % CI: -4.95, -1.99**	−1.52 (2.58); 95 % CI: -2.59, -0.46*	0.03

* = *p* < 0.01; ** = *p* < 0.001 within group comparison

**Table 5:** Serum testosterone level (ng/dL)

	Treatment group	Placebo group	Between group comparison
	Mean (SD)	Mean (SD)	(*p*-values)
Sample size (n)	*n* = 25	*n* = 25	
Pre intervention	630.45 (231.88)	675.12 (157.02)	0.43
Post intervention	726.64 (171.55)**	693.12 (115.04)	0.42
Change	96.19 (100.14); 95 % CI: 54.86, 137.53**	18.00 (81.94); 95 % CI: -15.83, 51.82	0.004

** = *p* < 0.001 within group comparison

**Table 6:** Muscle recovery: increase of serum creatine kinase from hour 24 to hour 48 after end of exercising (U/L)

	Treatment group	Placebo group	Between group comparison
	Mean (SD)	Mean (SD)	(*p*-values)
Sample size (n)	*n* = 25	*n* = 25	
Pre intervention	1478.88 (239.60)	1406.52 (264.45)	0.31
Post intervention	16.20 (9.47)**	99.04 (16.77)**	<0.0001
Change	−1462.68 (233.57); 95 % CI: -1559.09,-1366.27**	−1307.48 (253.54); 95 % CI: -1412.14, -1202.82**	0.03

** = *p* < 0.001 within group comparison

### Primary efficacy measure: muscle strength

**Fig. 2:**
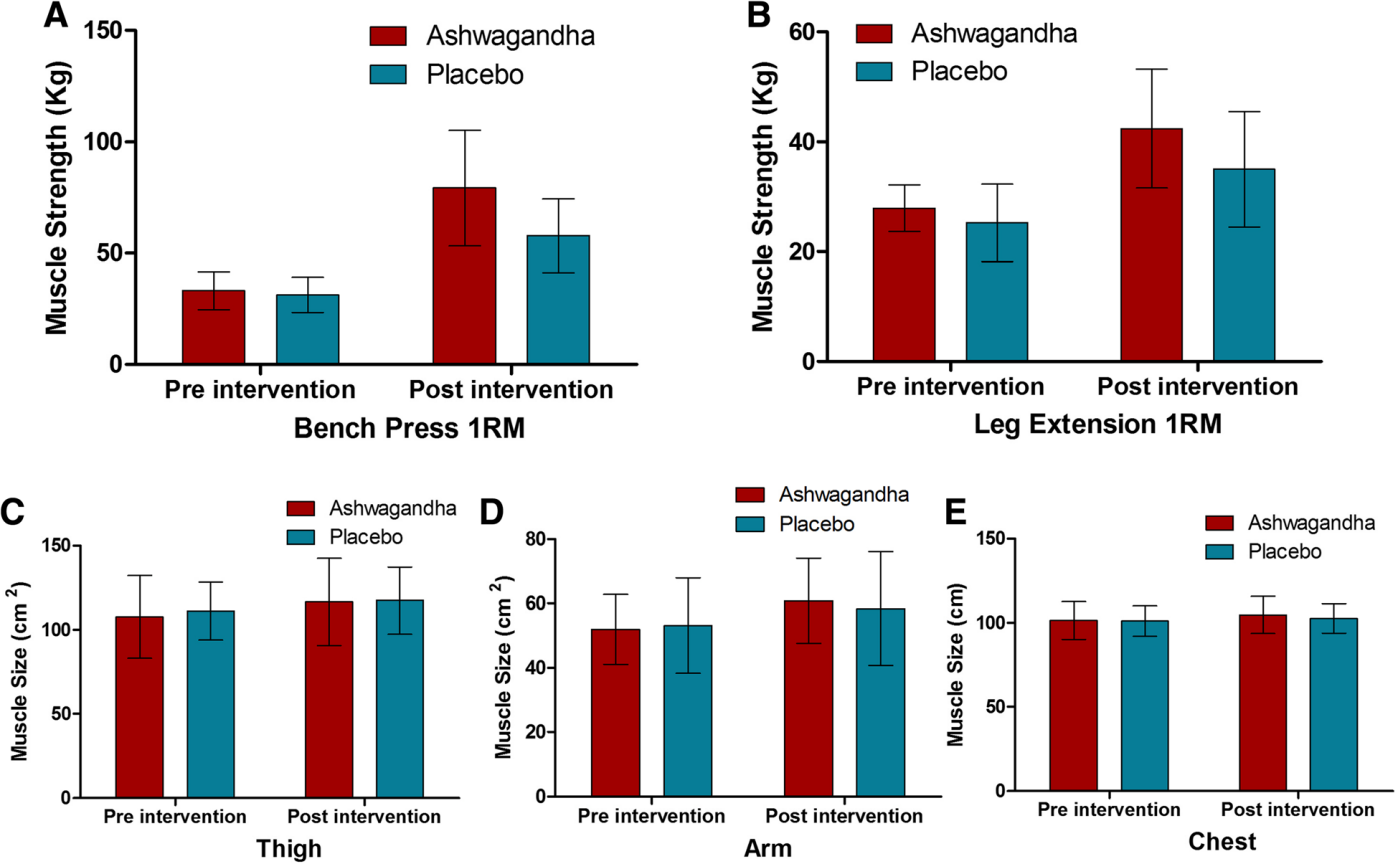
The effect of Ashwagandha treatment in muscle strength [**a** bench press 1 RM, **b** leg extension 1RM] and muscle size in **c** thigh, **d** arm and **e** Chest

There was a significant increase in muscle strength and muscle size in both the ashwagandha group and the placebo group, for both the upper and lower body. This is unsurprising because both group engaged in resistance training. The focal question is whether the adaptation is greater under ashwagandha supplementation. Table 2 and Fig. 2 show that the increases in muscle strength were statistically significantly greater in the ashwagandha group than in the placebo group, for the upper body (Placebo: 26.42 kg, 95 % CI, 19.52, 33.32 vs. Ashwagandha: 46.05 kg, 95 % CI 36.56, 55.54; *p* = 0.001) and the lower body (Placebo: 9.77 kg, 95 % CI, 7.18, 12.35 vs. Ashwagandha: 14.50 kg, 95 % 10.76, 18.23; *p* = 0.04).

### Secondary efficacy measures

#### Anthropometry

*Muscle Size:* For muscle size (Table 3; Fig. 2), the increases are significantly greater in the ashwagandha group than the placebo group in the arm (Placebo: 5.30 cm^2^, 95 % CI, 3.34,7.25 vs. Ashwagandha: 8.89 cm^2^, 95 % 6.95,10.84; *p* = 0.01) and chest (Placebo: 1.43 cm, 95 % CI, 0.83, 2.02 vs. Ashwagandha: 3.37 cm, 95 % CI, 2.59, 4.15; *p* < 0.001) but not in the thighs (Placebo: 6.22 cm^2^, 95 % CI, 2.61, 9.84 vs. Ashwagandha: 8.71 cm^2^, 95 % CI, 4.56, 12.87; *p* = 0.36).

*Body composition:* Table 4 shows that body fat percentages declined in both groups over the 8 week study, with the fat percentage decrease being significantly greater among subjects in the ashwagandha group as compared to the placebo group (Placebo: 1.52 %, 95 % CI, 0.46, 2.59, vs. Ashwagandha: 3.47 %, 95 % CI, 1.99, 4.95; *p* = 0.03).

#### Serum testosterone

**Fig. 3:**
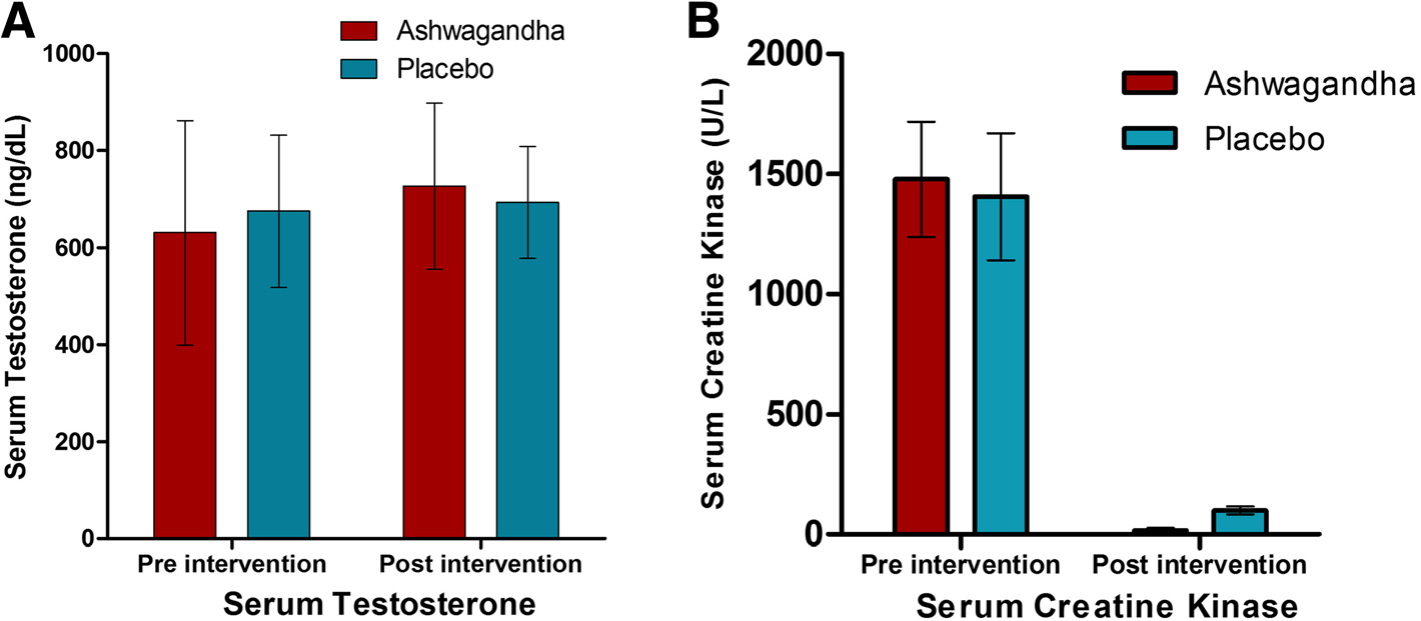
The effect of Ashwagandha treatment in **a** serum testosterone and **b** serum creatine kinase level

Over the eight weeks, there was a significant increase in testosterone level in the ashwagandha treatment group relative to the placebo group (Table 5; Fig. 3). The increase in testosterone level was significantly greater with ashwagandha supplementation than with the placebo (Placebo: 18.00 ng/dL, 95 % CI, -15.83, 51.82 vs. Ashwagandha: 96.19 ng/dL, 95 % CI, 54.86, 137.53; *p* = 0.004). While the mean post-intervention level was notably higher in the ashwagandha group than in the placebo group (726 versus 693), the numbers are not detectable as statistically significantly different, very likely because the across-subject variance is high.

#### Muscle recovery

Recall that the level of recovery from exercise-induced muscle damage is assessed through the increase in level of serum creatine kinase from Hour 24 to Hour 48 after the end of the resistance training session. A smaller increase in this muscle protein in the blood stream corresponds to faster muscle tissue repair, which in turn corresponds to greater recovery. Table 6 and Fig. 3 show how this metric varied across groups and over time. It is important to keep in mind that smaller numbers are to be interpreted as better recovery. What is striking is that recovery was dramatically better after 8 weeks of resistance training, in both the ashwagandha group and the placebo group, likely because of muscle tissue getting accustomed to the training regimen and developing greater integrity to resist any damage. Comparing the ashwagandha group and the placebo group, the results showed that recovery is substantially higher in the ashwagandha group than in the placebo group (Placebo: 1307.48 U/L, 95 % CI, -1202.82, 1412.14 vs. Ashwagandha: 1462.68 U/L, 95 % CI, 1366.27, 1559.09; *p* = 0.03).

### Tolerability

No serious side effects were reported by subjects in either group. All subjects rated tolerability as either “good” or “excellent” on the PGATT form. There was no statistically significant difference in PGATT scores between the 2 groups.

## Discussion

This is the first research paper that we know of that studies ashwagandha as an adjuvant to resistance training programs. Because subjects in both the ashwagandha group and the placebo group engaged in resistance training, we would expect to see a substantial degree of improvement in muscle-related parameters in both groups, and indeed we did. These findings are consistent with numerous studies which have measured adaptation to strength training programs in the absence of any supplementation [33]. The focal question that we sought to examine in this research is related to whether ashwagandha supplementation would magnify these adaptations. The adaptations were found to be statistically significantly greater, at a *p*-value threshold of 0.05, with ashwagandha supplementation than under placebo for all parameters (muscle strength, muscle size and body fat percentage, testosterone, and muscle recovery) except for thigh muscle size, though some effects were marginal. Increased recovery from muscle damage has the practical implication that it allows one to resume resistance training more quickly, thereby increase the volume of training per unit time and thereby potentially achieve greater gains per unit time. Our basic results are consistent with the findings of previous human studies with ashwagandha [8, 9, 28, 29, 52] in demonstrating gains in muscle strength, body composition and testosterone, though these other studies do not follow these parameters all in a single clinical trial or in conjunction with a resistance training program. The ashwagandha extract was tolerated very well at the study dose with no side effects reported. This good safety profile of ashwagandha is consistent with reports from previous studies [8, 9, 28, 29, 52].

There are also studies considering the use of other adaptogenic herbs like *Rhodiola rosea*, *Eleutherococcus senticosus*, *Schisandra chinensis* and ginseng toward physical performance. One study [53] gave evidence that *Rhodiola rosea* supplementation can improve endurance and reduce time to exhaustion. A review of Russian research [15] identifies human studies that show improved physical and mental performance from Schisandra supplementation. It is suggested that [15] Schisandra supplementation can help elite athletes adapt to high physical intensities. More study of the use of adaptogen herbs in the aid of muscle strength and recovery is needed.

There are several mechanisms of action that could have contributed to the positive effects of ashwagandha supplementation on resistance training and performance improvements in this study. These can be viewed from two perspectives: muscle development and muscle recovery.

### Muscle development

The ability to lift weights is a function of (a) muscle size, (b) energy production and (c) the nervous system’s ability to recruit muscles and coordinate them to generate the required force. Muscle size is a function of muscle growth, which is affected by two of ashwagandha’s effects: (i) increase in testosterone, which leads to muscle growth and (ii) decrease in the levels of cortisol, which as a catabolic agent detracts from muscle mass. In terms of energy production, ashwagandha (i) can have beneficial effects on mitochondrial energy levels and functioning and reduce the activity of the Mg2 + -dependent ATPase enzyme responsible for the breakdown of ATP [54], and (ii) can increase creatine levels that can in turn lead to ATP generation [8]. Finally, the effects of ashwagandha on the nervous system as anti-anxiety agent and in promoting focus and concentration [28] may translate to better coordination and recruitment of muscles. The reason for the lack of an effect on thigh size is not clear. Longer term studies are needed to shed light on this, as are studies looking at markers in the local environment of these muscles to rule out any biochemical anomalies as contributing factors.

### Muscle recovery

In the present study, more rapid recovery from muscle damage under supplementation with ashwagandha was demonstrated by monitoring creatine kinase. The faster recovery could be due to a number of mechanisms, or more likely their synergistic effects, as mediated by the various extract components, such as antioxidant effects to combat free radical damage both at the muscle and central nervous system levels, anti-inflammatory and analgesic effects and reduction in lactic acid and blood urea nitrogen [4–6]. In that vein, muscle soreness is a common occurrence following exercise for those less accustomed to physical activity. Delayed onset muscle soreness (DOMS) presents between 24 and 48 h after exercise as tenderness to palpation, and/or movement accompanied by decreases in flexibility and maximal voluntary force production. This soreness can inhibit full and proper exercise. Thus, a reduction in DOMS as a consequence of ashwagandha’s effect on reduced muscle injury would counteract this negative consequence.

## Conclusion

This study confirms previous data regarding the adaptogenic properties of ashwagandha and suggests it might be a useful adjunct to strength training. This study has the following limitations which should lead us to interpret the findings with some caution: the subjects are untrained and moderately young, the sample size of 50 is not large and the study period is of duration only 8 weeks. Research studying the possible beneficial effects of ashwagandha needs to be conducted for longer periods of time and for different populations including females and older adults of both genders.
